# News and Events of the Week

**Published:** 1910-12-10

**Authors:** 


### News and Events of the Week.

John Lewis Prichard, M.D., L.R.C.P., L.R.C.S., of 2 St. Helens Crescent, Swansea, Glamorgan, who died on September 28, has left estate valued at £29,628 gross and £25,091 net.

An investigation carried on by the Commissioner of Accounts of New York has revealed the fact that in that city there exists a considerable trade in condemned eggs, familiarly known as "rots and spots," which after being declared bad have been disposed of to bakers' supply stores.

Commenting on the political defeat of Sir William Collins, M.D., in West St. Pancras on Monday, the *Times* said: "In West St. Pancras Mr. Felix Cassel, the Unionist, defeated Sir William Collins by eight votes after a recount had been taken. In January, oddly enough, Sir William Collins retained the seat by ten votes after a recount. . . . Unionists, however, . . . will be the first to admit that Sir William Collins has done much useful work in Parliament, where he was a popular member." Our readers have special means of knowing the truth of this statement.

The first congress of French medical journalists, organised by the Association des Journalistes Médicaux Français, will be held at Paris on March 23, 1911, at 9 a.m. in the hall of the Hôtel des Sociétiés Savantes, rue Danton. The agenda includes, among other subjects of interest, discussions on the copyright of medical articles, and on the usurpation of the title of doctor. A dinner will be given to members of the Congress at the conclusion of the proceedings. All medical journalists are eligible for membership. Particulars may be obtained on application to the general secretary, Dr. Cabanés, 9 rue de Poissy.

Mr. Roger Hughes, of High Street, Bala, Merioneth, retired surgeon, who died on July 30, left estate valued at £12,001 gross, with net personalty £9,684.

The *London Gazette* states that the King has granted to Mr. Eben Henry Edwards, M.B., C.M., his Royal licence to accept and wear the Insignia of the Third Class of the First Division of the Imperial Chinese Order of the Double Dragon, conferred upon him by the Emperor of China.

Dr. Newmann, Chief Medical Officer of the Board of Education, has issued his report for 1909. It deals with the first year in which the results of the medical examination of the children attending elementary schools in England and Wales have been ascertainable and comparable. It is of the greatest importance, and we shall hope soon to deal with it at the length it demands.

We congratulate Professor A. Gilbert, of the Paris Faculty of Medicine, on the appearance of the first number of *Paris Médical*, a weekly clinical journal which the professor, aided by a staff of well-known writers, edits. The thirty pages of this interesting new publication are crammed with information, original and derived, and there is no doubt that Professor Gilbert will succeed in making *Paris Médical* in France what The Hospital is in England—the general practitioner's paper *par excellence*. The articles in the first issue are thoroughly practical, well illustrated, and printed in clear, bold type on good paper. The paper will deal chiefly with French medicine in its various aspects, and promises to be one of the most useful of the Continental weeklies for reference purposes. The annual subscription (outside France) is 15 francs, and the publishers are Messrs. J. B. Baillière et Fils, 19 rue Hautefeuille, Paris.

The annual meeting of Presidents of the League of Mercy will be held on Monday, December 19, at 4 p.m., in the Board Room of the Middlesex Hospital, the Right Hon. Lord Farquhar, G.C.V.O., in the chair.

The King has been graciously pleased to sanction the following among other promotions in and appointments to the Order of the Hospital of St. John of Jerusalem in England : As Knight of Grace, Sylvanus Glanville Morris, Esq., M.D.

Mrs. Mary Baker Eddy, the Christian Science leader, died early on Sunday, December 4, telegraphs the *Times* New York correspondent, at her Boston home. Nothing definite is known as to her successor. The Church trustees are expected to take action after the funeral.

Sir Norman Baker, the Lieutenant-Governor of Bengal, presided, says the *Times* correspondent, at a meeting of the General Committee of the Bengal King Edward Memorial Fund at Belvedere, at which it was unanimously resolved, on the motion of the Maharajah of Durbhanga, to lay aside one and a half lacs of rupees (£10,000) for an equestrian statue to his late Majesty on the best available site on the Calcutta Maidan. The remainder of the fund will be devoted to the promotion of medical research, hospitals, and kindred objects.

The Animal Defence and Anti-Vivisection Society held at Caxton Hall, Westminster, a protest meeting against the candidature of Sir Victor Horsley for the representation of London University "and the attempts being made to identify vivisection with education." Dr. Forbes Winslow, who presided, said it was not a question of personal hostility, but of principle. The following resolution was then carried amid cheers : "That this meeting protests against the election to Parliament of any vivisector," and urged that they should oppose to the utmost the return of Sir Victor Horsley, who had, according to his own evidence given before the Royal Commission on Vivisection, performed over 3,000 experiments on living animals.

The annual dinner of the Royal Society of Medicine took place this year at the Connaught Rooms. Sir Henry Morris, the president, occupied the chair, and the company included the Lord Mayor, Lord Ilkeston, Lord Blyth, Lord Justice Fletcher Moulton, Mr. Justice Lush, Sir William Church, Sir Joseph Dimsdale, Sir Almeric FitzRoy, Sir R. Douglas Powell, Sir William Treloar, the Vice-Chancellor of the University of London (Professor M. J. M. Hill, F.R.S.), the Chairman of the London County Council, Surgeon-General Branfoot (President of the Medical Board of the India Office), Mr. Charters Symonds (President of the Medical Society), Sir Thomas Barlow, Mr. H. T. Butlin, Sir James Reid, Sir James Porter (Director-General Medical Department of the Navy), Sir Alfred Pearce Gould, Dr. Squire Sprigge, Sir Lauder Brunton, F.R.S., Sir Shirley Murphy, Sir John Broadbent, Colonel Sir David Bruce, and Mr. J. Y. W. MacAlister. The President said that a notable event of the past year was the admission of women as Fellows of the Society. There were to-day 1,050 women who had passed through the London School of Medicine, and of that number he understood that 800 had taken a degree at the University of London. There were 855 women practitioners on the Register. The Royal Society of Medicine would like to take a share in the investigation of tropical disease.

The medical superintendent of Croydon Infirmary would welcome help on behalf of Mrs. Godfrey, of Beulah Grove, West Croydon, who on November 6 gave birth to triplets (all of whom have survived), and who has received the King's bounty. She is in very poor circumstances, her husband being out of work.

The attractive Christmas number of *Truth* is "The Crankiad," which mainly consists of a rhyming chronicle of various fads and faddists. The faculty will appreciate the fun as much as lay readers will, and we recommend practitioners who desire a dose of hearty humour during the festive season to lay in a copy of "The Crankiad."

At the request of a large number of members of the medical profession who use motor-cars for the purposes of their profession the Motor Union has addressed a letter to the Treasury with reference to the use of motor-cars on which rebate has been allowed and the following reply has been received by the assistant secretary of the Union : "Treasury Chambers, November 18, 1910. Sir,—In reply to your letter of the 10th inst., I am directed by the Lords Commissioners of his Majesty's Treasury to state that, in accordance with Section 86 (4) of the Finance (1909-10) Act, 1910, it is for the County Council to satisfy itself on the question of fact whether a motor-car in respect of which a medical practitioner claims relief of half the rate of duty payable is kept for the purpose of his profession. Provided that the Council is satisfied on this question, my Lords are of opinion that the medical practitioner is entitled to the allowance, although he may at times use the car for other purposes.—I am, sir, your obedient servant, J. H. Murray."

